# Near-Complete Genome Sequence of White Spot Syndrome Virus Infecting Cultivated Shrimp (Penaeus vannamei) in Peru

**DOI:** 10.1128/mra.00040-23

**Published:** 2023-05-09

**Authors:** Luis Alberto Salcedo-Mejía, Stephanie Tapia-Chirinos, Joel Atarama-Orejuela, Sandra Grabiel, Eduard Villalobos, Muriel Gómez-Sánchez, Rodolfo Velazco-Peña

**Affiliations:** a Laboratorio de Sanidad Acuícola, Sede Tumbes, Organismo Nacional de Sanidad Pesquera (SANIPES), Lima, Peru; Queens College Department of Biology

## Abstract

White spot syndrome virus (WSSV) infects a broad range of aquatic animals, including the shrimp Penaeus vannamei. In this study, we report one genome sequence of WSSV present in shrimp on the north coast of Peru.

## ANNOUNCEMENT

White spot syndrome is the most severe threat to all cultivated species of shrimp worldwide (1). In 1999, white spot syndrome virus (WSSV) produced an important outbreak in the species Penaeus vannamei, Penaeus stylirostris, and Penaeus californiensis in the delta of the Tumbes River (north Peru), which had a heavy economic impact on local aquiculture (2). This virus has an approximately 300-kb, double-stranded circular DNA genome (genus *Whispovirus*, family *Nimaviridae*) (1).

We sequenced a WSSV genome from a sample from a shrimp farm pond collected while monitoring diseases of farmed shrimp (*P. vannamei*) in Tumbes, Peru; the specimen showed white spots on the exoskeleton, but no mortality was observed. Pleopods were collected and homogenized by pestle. Total DNA was isolated using the NucleoSpin tissue kit (Macherey-Nagel, Germany), and molecular detection was conducted by real-time PCR following the recommendations of the World Organization for Animal Health (OIE) (3). WSSV was detected with a *C_T_* (cycle threshold) of 22.5 (*vp664* gene). A library was generated using the Nextera DNA Flex library prep kit. Sequencing was performed on the Illumina NovaSeq 6000 system (2 × 150-bp, paired-end format). In all, 536,694,682 raw reads were obtained. The shrimp reads were not removed prior to mapping. A total of 15.82% (51,337,280 reads) were mapped to WSSV.

Nineteen contigs were obtained; of these, only 13 ordered contigs were considered and assembled into a linear genome using the Contiguator2 program v2.7.5. It should be noted that the contigs that were not included may have been too small or contained repetitive regions. The gaps were filled with N using a reference genome at NCBI (GenBank accession number AF332093.3).

The Illumina reads were trimmed using Trimmomatic v0.39 and mapped to reference genomes submitted under GenBank accession numbers AF332093.3 and NC_003225.3/KT995472.1 using the Bowtie2 v2.3.5.1 tool (4). The shrimp reads were not removed prior to mapping. The MEGAHIT program v1.2.9 was used for *de novo* assembly, and 19 contigs were identified and aligned with the reference genomes using the MAUVE v2.4.0 program (5, 6). The 13 ordered contigs were assembled using the CONTIGuator v2.7.5 program (7) into a linear genome, the gaps were filled with N, and the reference genome AF332093.3 was used as a template. Open reading frame (ORF) determination and genome annotation were conducted using Prokka v1.14 (8), and the genome was aligned with 18 complete WSSV genomes (AF332093.3, MN840357.1, NC_003225.3/KT995472.1, MH090824.1, KY827813.1, KT995470.1, KT995471.1, JX515788.1, MG432475.1, MG432477.1, MG432476.1, MG432478.1, AF440570.1, AF369029.2, KU216744.2, MG264599.1, MF768985.1, and KX686117.1) using MAFFT software v7.310 (9).

The WSSV genome comprises approximately 278,607 bp, with a GC content of 40.94% and a coverage depth of >20,000×. In total, 157 ORFs were observed. The genome is closely related to a WSSV genome found in Ecuador (GenBank accession number MH090824) and more distantly related to a strain from the United States (MN840357).

The average nucleotide identities (EZBioCloud; https://www.ezbiocloud.net/tools/ani) between our Peruvian genome and the ICTV reference genome, U.S. genome, and Ecuadorian genome are 99.65%, 99.52%, and 99.46%, respectively.

**FIG 1:**
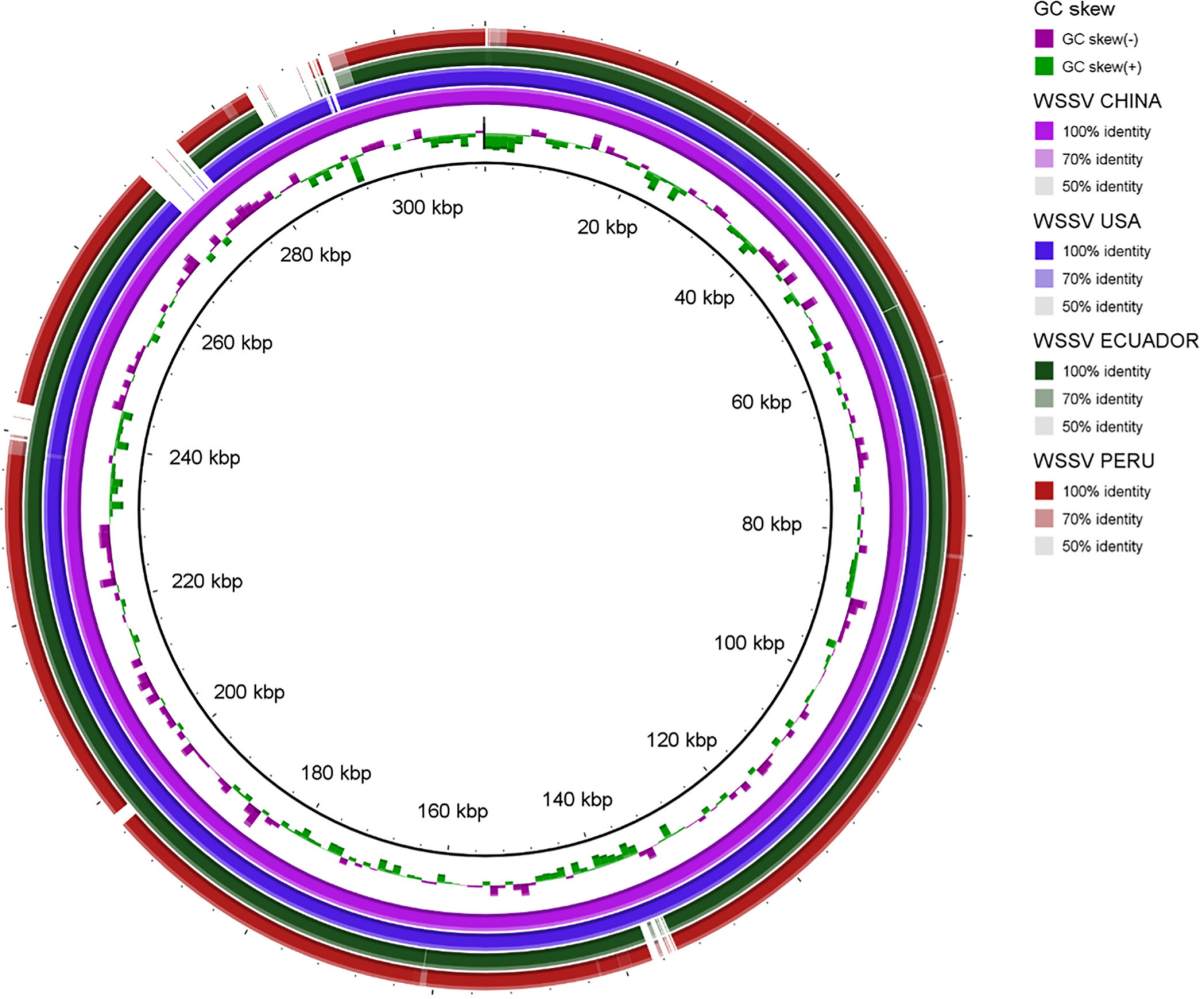
BLAST comparisons of 3 other published WSSV genomes (submitted under GenBank accession numbers MH090824 [Ecuador], MN840357 [USA], and AF332093 [China]) with the near-complete WSSV genome in this study from Peru (OM350393.2) (created using BLAST Ring Image Generator).

We observed deletions relative to the reference genome NC_003225.3 (Fig. 1). The first deletion (~1,300 bp) contained fragments of VP62 and VP39 ORFs. The second deletion (~2,700 bp) contained fragments of VP41A, VP41B, and VP52A ORFs. These four ORFs encode envelope proteins. It should be noted that WSSV possesses several repetitive regions, and the methodology using Illumina reads may have been insufficient for completing the entire genome. Therefore, it is possible that some regions of the genome were not fully assembled. More research should be conducted to evaluate the pathogenic potential of strains present on the coast of Tumbes, Peru.

### Data availability.

The sequence was deposited at GenBank under accession number OM350393.2. The raw sequence data are available under SRA accession number PRJNA848017.
